# 

**Published:** 1877-06

**Authors:** 


### INDEX TO VOLUME XXXIV.

**A**bsorption of Mercury, - - - - - - 574
Addison's disease, - - - - - - 456
Ages at which marriages take place in each sex, - - - 269
Albuminuria and pregnancy, - - - - - 470
Allport, Dr. F., Corn sweat, - - - - - - 133
American medical association, - - - - - 445
American medical bi-weekly, - - - - - 467
American medical weekly, - - - - - 271
Anatomist, The oil painting, - - - - - - 466
Andrews, Dr. E., Mortality of surgical operations, - - 23, 121
Anhidrotics, - - - - - - - - 286
Aniline causing disorders. - - - - - - 84
Anomalies of the conduction of sounds in the ear, - - 382
Anthony, Diphtheria, - - - - - - 374
Antidotes of arsenic, - - - - - - 185
Antiseptic dressing, - - - - - - 87
Antiseptic property of eserine and atropia, - - - 579
Anti-pruritic, - - - - - - - 186
Anti-pyretic action, Principles of, - - - - 285
Aphonia, - - - - - - 375, 496
Archambault, Umbilical hernia, - - - - 378
Arlt, Blepharorhaphia, - - - - - - 189
Army, Annual report, - - - - - - 68
Ascarides *vs.* Asteroides, - - - - - - 270
Ascites, Treatment, - - - - - - - 280
Attfield, F., Chemistry, - - - - - - 69
Atheromatous degeneration, - - - - - - 431
Athetosis, - - - - - - - 162
Atropiae sulphas for otitis, - - - - - 104
Atropia for epilepsy, - - - - - - 573
**B**aker, Dr. S., Eclampsia, - - - - - - 213
Balanitis, - - - - - - - 190
Balfour, Antiseptic dressing, - - - - - 87
Balsam of Peru, - - - - - - 183
Banga, Posture for comp'd fracture, - - - - 485
Barnes, Dr. T. K., Medical history, - - - - 257
Bartholow, Dr. L., Materia medica - - - - - 447
Bartlett, Dr. I., Silk worm epidemic, - - - - 108
Batty, Dr. R., Alabama association, - - - - 440
Bed sores, to prevent, - - - - - - - 89

Bernhardt, Dr. M., Athetosis, - - - - - 162
Berthomier, Fractures in children, - - - - 567
Bertolet, Regeneration of nerves, - - - - - 85
Beverly, Aphonia, - - - - - - - 375
Bill for an act to regulate the practice of medicine, - - 267
Billroth, Prof., - - - - - - - 268
Bismuth, Liquid or elixir, - - - - - - 186
Blake, Intestinal obstruction, - - - - - 277
Blepharorhaphia medialis, - - - - - - 189
Boracic acid for wound dressing, - - - - 177
Bridge, Dr. N., Scirrhus, - - - - - 330
" Nasal irrigator, - - - - - 444
Bromide of camphor, - - - - - - 287
" of lithium, - - - - - - 186
" of potassium, - - - - 92, 185
Bromohydrate of conia - - - - - - 90
Brown, Salicylate of quinine, - - - - - 187
Bruen, Jaborandi, - - - - - - 576
Brunet, Dr., Version practiced in Polynesia, - - - 80
Buck, Dr. G., Reparative surgery, - - - - - 134
Buckley, Ergot *vs.* purpura, - - - - - 483
Bulkey, Anti-pruritic remedy, - - - - - 186
Busch, Necrotic process in bone, - - - - 278
Byford, W. H., Bloody tumor, - - - - - 289
Calabar, Use of, - - - - - - - 577
Calabar bean, galactagogue, - - - - - 90
Calculi arrested in the urethra, - - - - - 177
Calderin, hymen imperferatum, - - - - - 282
Cancer, villous, - - - - - - - 316
" encephaloid, - - - - - - 323
" of the lungs, - - - - - - - 430
Cane, Boracic acid, - - - - - - 177
Carbuncle, Treatment of, - - - - - - 568
Century of American medicine, A, - - - - - 252
Cerebral exhaustion, - - - - - - - 275
Cervix uteri, aspirating movements of, - - - - 282
Charcot, Preston, Sclerosis in patches, - - - - 332
Chassagny, Dr., Premature labor, - - - - - 471
Chemistry, by Attfield, - - - - - - 69
Chicago medical college, Graduates, - - - - - 366
Chicago medical society, - - - - - - 466
Chicago medical press association, - - - 76, 266, 365
Childbearing increasing ear diseases, - - - - 47
Chloral hydrate, - - - - - - 187, 234
Chorion, Hydatidiform degeneration of, - - - - 52
Chylus urine. - - - - - - - - 84
Cialtaglia, Diphtheria, - - - - - - 174

Classification of diseases, - - - - - - 200
Cleansing the nasal passages, - - - - - - 385
Cleland, Dissections, - - - - - - 260
Cod liver oil, emulsion of, - - - - - - 185
Cohnheim, Metastases, - - - - - - 474
Comegys, Ergot in diarrhœa, - - - - - - 91
Conium, - - - - - - 91
Cook county hospital, - - - - - 78, 426
Cooley, Dr. O., Death of, - - - - - - 161
Cormack, Diphtheria, - - - - - - 474
Corn sweat, - - - - - - 132
Corr, Sleeping disease, - - - - - - 376
Courbis, Luxation of the foot, - - - - 181
Cras, Fracture of the cranium, - - - - - - 180
Crandall, Hæmorrhage, - - - - - - 184
Critchett, Keratatis by vaccination, - - - - 579
Cumberland county medical society, - - - 78
Cyclopedia of medicine, Ziemssen's, - - - 137, 140
Cystic degeneration of inferior maxilla, - - - 430
Curtis Optical capacity of microscopes, - - - 526
Cystitis, Irrigation in, - - - - - - 569
Darvosky, Syphilis, - - - - - - 92, 190
Davis, Dr. F. H., Sponge holder, - - - - - 22
Davis, Dr. N. S., State of medical science, - - - 399
Day, Dr. E., Mola, - - - - - - 411
Denig, R. M., Quinine idiosyncrasy, - - - - 479
Dental anatomy, Guide to, - - - - - - 70
Depaul, Spondylozema, - - - - - - 473
Desault, - - - - - - 270
Detroit Medical journal, - - - - - - 271
Diabetes mellitus, - - - - - - 167
Dibrell, Treatment of carbuncle, - - - - - 568
Diphtheria, - - - - 174, 209, 285, 374
Di Pinciro, Rupture of the bag, - - - - - 283
Disinfectant, - - - - - - - 235, 286
Dissections, Directory for, - - - - - - 260
Dœring, Ulcers, - - - - - - 90
Dominik, Gunshot wounds, - - - - - - 379
Dowell, Dr. G., - - - - - . 359
Dumreicher, Treatment of wounds, - - - - - 570
Earle, Dr. Ch. W., Degeneration of the chorion, - - - 52
Eclampsia, Puerperal, - - - - - - 213
Eczema, Infantile, - - - - - - 90
Editorial, - - - - - - - 57, 272
Edmiston, Dr. D. W., Salicylic acid, - - - - 132
Eisenbockius, D. F. B., Scarlet fever, - - - - 310
Ellis, Dr. A. N., Atropia, - - - - - - 104

Ellis, Dr. A. N., Pleuresy, - - - - - 273
Embolism, Cerebral, - - - - - 49
" Pulmonary artery, - - - - - 178
Enballometer, - - - - - - 314
Empyema, Operation for, - - - - - 505
Epilepsy, Atropia for, - - - - - 573
Epistaxis, - - - - - - 412
Epithelioma, - - - - - 41, 413
Ergot in spleen enlargement, - - - - 39
" in diarrhœa, - - - - - - 91
" for purpura, - - - - - 483
Eserine, on glaucomatous eyes, - - - - 576
Eserine, Use of, - - - - - - 578
Esmarch's bandage, Hemorrhage after, - - - - 572
Fever, Lectures on, - - - - - 457
Fenn, Dr. C. F., Urinary fistula, - - - - 350
Ferguson, Death of, - - - - - 363
Fibro-cystoma, Uterine, - - - - - - 97
Fifth international ophthalmic congress, - - - 458
First intention, Means promoting, - - - - 408
Fitz, Rupture of the œsophagus, - - - - 375
Flint, Jr., Dr. A., Physiology, - - - - - 451
Foreign bodies in the eye, - - - 148, 149, 189
" in the œsophagus, - - - - - 178
Fothergill, Anhidrotics, - - - - - 286
Fox, Dr., Skin diseases, - - - - - 260
Fracture of the cranium, - - - - 180, 182
Fracture of the patella, - - - - - - 280
Fracture of the elbow in children, - - - - 567
Fredet, Aniline manufacturing causing disorders, - - 84
Freer, Dr. J. W., Obituary, - - - - 461
Frothingham, Pulsating tumor, - - - - 377
Gaetjens, Dr. H. O., Necrology, - - - - 362
Galactagogue, Calabar bean as, - - - - - 90
Gallicier, Cervix' uteri aspirating movement, - - - 282
Genital irritation, - - - - - 170
Gerhardt, Sclerosis, - - - - - - 173
Gibbons, H. Diphtheria, - - - - - 285, 361
Githens, Dr., Viability of a six month's fœtus, - - - 469
Glaucoma, Acute, - - - - - 188
Glycerole of nitrate of bismuth, - - - - 185
Goll, Dr. J., Necrology, - - - - - 362
Gore, Dr. A. A., Medical history, - - - - 146
Goss, Monobromide of camphor, - - - - 91
Grant, Tetanus, - - - - - . - 276
" Fracture of patella, - - - - 280
Green, Dr. T. H., Morbid anatomy, - - - - - 145

Greenhow, Dr. E. H., Addison's disease, - - - - 456
Griffith, Phymosis, - - - - - - 176
Grossheim, Gunshot wounds, - - - - - 378
Gruber, Prof., Anomalies of the conduction of sounds, - 382
Guerin, Vaccinal syphilis, - - - - - - 93
Guy's hospital reports, 1876, - - - - - 59
Gunshot wounds, - - - - - 378, 379
Hamburger, Absorption of mercurials, - - - 574
Hæmorrhage, from a varix, - - - - - 183
" in coitu, - - - - - - 184
" Treatment with hot water, - - - - 281
Hæmorrhoids, Treatment, - - - - 247, 351, 435
Hagenbuch, Dr. A. W., Diphtheria, - - - - 209
Hamilton, Dr., Conium, - - - - - 91
" Genital irritation, - - - - 170, 465
Hammond, Dr. W. A., Diseases of nervous system, - - 68
Harelip, - - - - - - 432
Harvey, - - - - - - - 269
Headache, Remedy for, - - - - - 573
Hildebrandt, Pneumonia, - - - - - - 274
History, Medical, of the West-American campaigns, - - 146
Hoadley, Dr. A. E., Leucocythæmia, - - - - 326
Hotz, Dr. F. C., Clinical reports, - - - - 148
Hunter, Dr. G., Puerperal fever, - - - - 369
Hyde, Dr. I. N., Preputial stenosis, - - - 295
Hydroadipsia, - - - - - - 473
Hymen imperforatum, - - - - - - 282
Hysterical Aphonia, - - - - - - 496
Illinois Charitable Eye and Ear Infirmary, - - - 467
Illinois State Medical Society, - - - - - 536
Illustrating Microscopical Observations, - - - 193
Impetigo, Infantile, - - - - - - 90
Ingals, E. F., Tympanitic resonance, - - - - 212
" Chloral, Death from - - - - - 234
" Enballometer, - - - - - - 314
" Improved operation for empyema, - - - 505
Ingerslev, Weight of newborn infants, - - - - 87
Inflammation of middle ear, - - - - - 380
Inflammatory process in bone, - - - - - 278
Insomnia, - - - - - - - 13
Intestinal obstruction, - - - - - - 277
Irrigation in chronic cystitis, - - - - - 569
Jaborandi in Bright's disease, - - - - - 576
Jackson, Constant irrigation in cystitis, - - - 569
Jenks, Viburnum primifolium, - - - - - 92
Johnson, C. B., Classification of diseases, - - - 200
Johnson, H. A., Thyrotomy, - - - - - 1

**K**eats, Disinfectant, - - - - - - - 286
Kinsman, Dr., Synopsis of obstetrical practice, - - 469
Klemm, Vocal cords, - - - - - - - 175
Knox, Dr. J. T., Insomnia, - - - - - 13
Keratitis by vaccination, - - - - - - 579
**L**acey, Dr. Th. B., Mortality of surgical operations, etc., - 23, 121
Landis, Dr. E. M., Harelip - - - - - - 432
Langenbeck. Dr. M., Necrosed nasal bones, etc., - - 377
Laqueur, Eserine for glaucoma, - - - - - 576
Laryngeal polypi, Removal of, - - - - - 478
Lasinski, Whooping-cough, - - - - - - 284
Lee, Dr. E. W., First intention, - - - - - 408
Leisham, Dr. W., Midwifery, - - - - - 258
Leucocythæmia, - - - - - - - 326
Lewis, Dr. C. T., Cancer in a child, - - - - 323
Ligation of the external iliac artery, - - - 249
Lime water, - - - - - - - - 90
Lipoma, - - - - - - - - 280
Liquor potassæ, - - - - - - - 89
Lockbridge, Remedy for headache, - - - - - 573
Longhi, Tincture of tayuya, - - - - - - 94
Luecke, Dr., Percussion of the bones, - - - 86
Luxation of the foot, - - - - - - - 181
Lyman, Cerebral embolism, - - - - - 49
" Scarlet Fever, - - - - - - 514
**M**agnus, Acute glaucoma, - - - - - 188
May, Antipyretic action, - - - - - - 285
Maish, Metric weights, - - - - - - 171
Malarial diseases, - - - - - - - 359
Martin, Foreign body in the eye, - - - - - - 189
" Albuminuria and pregnancy, - - - - 470
Massari, Embolism, - - - - - - - 178
Massachusetts eye and ear infirmary report, - - - 271
Materia medica, Treatise on, - - - - - 447
Maunder, Dr. F. C., Surgery of the arteries, - - - 71
McFlory, Hydroadipsia, - - - - - - 473
Meadows, Dr. A., Midwifery, - - - - - - 285
Medical association of Alabama, - - - - - 440
Medical history, Contributions to, - - - - - 146
" of the war of rebellion, - - - - - 257
Meyer, Dr. I. H., Skin grafting, - - - - - 320
" Tuberculosis, - - - - - - 329, 428, 347
" Scarlatina, - - - - - - - 426
Membrana tympani, artificial, - - - - - 382
Metastases, - - - - - - - 474
Metric weights, - - - - - - - 171
Metzger, Tuberculosis, - - - - - - - 376

Michael, Nitrite of Amyl, - - - - - - 382
Michigan state board of health, - - - 80, 364, 466
Michigan university, - - - - - - - 466
Microscope, Optical capacity of, - - - - - 526
Midwifery, text-books, - - - - - - 258
Miller, Ergot in spleen enlargement, - - - 89
Mitchell, Regeneration of nerves, - - - - 85
" Spasm, - - - - - - - 167
Monobromide of camphor, - - - - - 91
Morgan, Chloral hydrate, - - - - - - 187
Mortality of surgical operations, etc., - - - 23, 121
Mosler, Diabetes mellitus, - - - - - - 167
Mourut, Bromohydrate of Conia, - - - - 90
**N**aevi materni, - - - - - - 274
Nasal irrigator, - - - - - - - 444
Nancude, Dr. Phlebitis, - - - - - 179
Necrosed nasal bones swallowed, - - - - 377
Nervous system, Diseases of, - - - - 69, 137
Nestle's food, - - - - - - - 175
Neumann, Hypodermic treatment of syphilis, - - - 95
Neurectomy nervi infraorbitalis, - - - - - 281
New York ophthalmic and aural institute, - - - 363
Nitrite of Amyl, - - - - - 186, 382
Nitrate of potassium, - - - - - - 310
Nose, Diseases of, - - - - - - 133
Norwood, Ascites, - - - - - - - 280
Notta, Dangers of pessary, - - - - - 88
**O**bstetric practice, - - - - - - - 534
Obituary: Dr. Cooley, - - - - - - 161
" Dr. H. O. Gaetjens, - - - - - - 362
" G.Goll, - - - - - - - 362
" Fergusson, - - - - - - - 363
" Dr. G. Buck, - - - - - - 363
" Dr. J. W. Freer, - - - - - - 461
Ophthalmic and otic memoranda, - - - - - 187
Owens, Dr. T. E., Hæmorrhoids, - - - - 247, 351, 435
**P**aracentesis of the pericardium, - - - - 373
Paralysis after diphtheria. - - - - 151, 474
Parotid gland extirpated, - - - - - - 178
Pathology and morbid anatomy, introduction to, - - 145
Peliosis rheumatica, - - - - - - - 276
Pemphigus neonatorum, - - - - - - 366
Percussion of the bones, - - - - - - 86
Pessary, Dangers of, - - - - - - 88
Peyrand, Bromide of potassium, - - - - - 92
Phlebitis, - - - - - - - 179
Phymosis, Cure of, - - - - - - - 176

Physiology, Human. - - - - - - 451
Pierce, Dr. F. M., Childbearing and deafness, - - - 47
Piper, Dr. R. U., Pictorial art etc., - - - - - 193
Playfair, Dr. W. S., Midwifery, - - - - - 258
Pleuritic effusion, - - - - - - 273
Pneumonia, Intermittent, - - - - - - 274
Polaillon, Hæmorrhage, - - - - - 183
Premature labor, - - - - - - - 471
Posture for compound fracture, - - - - - 485
Premature rupture of the bag, - - - - - 283
Preputial Stenosis, - - - - - - 295
Present state of medical science, - - - - 399
Puerperal fever, - - - - - - - 369
Pulsating tumor, - - - - - - - 377
Pulsatilla, - - - - - - - 576
Purpura, - - - - - - - - 483
Putnam, Nestle's food, - - - - - - 175
Pygomelia, Living specimen of, - - - - 81
**Q**uarterly journal of inebriety, - - - - - 363
Quinine, Idiosyncrasy, - - - - - - 479
" Salicylate of, - - - - - - 187
**R**ectal dilator, New, - - - - - - - 475
Regeneration of nerves, - - - - - - 85
Reparative surgery, - - - - - - - 134
Report, annual, of the surgical general U. S. A., 1876, - 68
Reports, Guy's hospital, - - - - - - 59
Riedinger, Hemorrhage after Esmarch's bandage, - - 572
Roberts, Dr. D. L., Midwifery, - - - - - 258
" Paracentesis, - - - - - - 373
Robin, Chylous urine, - - - - - - 84
Roosa, Dr. D. B. St. J., Syphilis of cochlea, - - - 190
Rouyer, Antidotes of arsenic, - - - - - - 185
Rupture of the œsophagus, - - - - - 375
Rush, Dr. B., Memoir of - - - - - - 367
Rush college clinics, - - - - - - 432, 531
**S**alicin, - - - - - - - - 188
Salicylic acid, Solvent for, - - - - - 131, 287
" Strong solution, - - - - - - 186
Salicylate of quinine, - - - - - - 187
Savage, Dr. H., Surgery of the pelvic organs, - - 356
Scarlatina, Complication, - - - - - - 426
Scarlet fever, - - - - - - 226, 310, 374
Scarlet fever and board of health, - - - - 514
Scirrhus of the bile duct, - - - - - 330
Sclerosis in patches, - - - - - - - 332
" Cerebro-spinal, - - - - - - 173
Schede, Treatment of varicose veins, - - - - 567

Schmidt-Rimpler, Antiseptic property, - - - - 579
Schweig, Cerebral exhaustion, - - - - - 275
Septicæmia, - - - - - - - - 369
Sewill, Dental anatomy, - - - - - - - 70
Sexton, S., Inflammation of the middle ear, - - - 380
Shall, Diptheria, - - - - - - - 89
Shortening of the limb after fracture of the femur, - - 477
Silk-worm, epidemic and, - - - - - - - 108
Skin diseases, - - - - - - - 260
Skin grafting, - - - - - - - 320
Skin, healthy, - - - - - - - 262
Sleeping disease, - - - - - - - 326
Slocum, Fracture of the skull, - - - - - 182
Spasm, Functional, - - - - - - - 167
Spondylizema, - - - - - - - 473
Spongeholder, Laryngeal, - - - - - 22
Squire, Nitrate of bismuth, - - - - - - 185
Staples, Dr. F., Uterine fibro-cystoma, - - - - 97
Stedman, Scarlatina, - - - - - - 374
Stevenson, Dr. S. H., on Dr. Freer, - - - - 442
Stilling, Lipoma, - - - - - - - 280
" Neurectomy, - - - - - - 281
Stinking feet, - - - - - - - 412
Stokes, Dr. W., on fever, - - - - - - 457
Sulphide of carbon, in ulcers, - - - - - 90
Sulphurous acid a disinfectant, - - - - - 286
Sulpho-carbolate of sodium, - - - - - 374
Surgery, etc.,of the female pelvic organs, - - - 356
" of the arteries, - - - - - - 71
Synopsis of obstetrical practice, - - - - 469
Syphilis, - - - - - 92, 93, 95, 190
Swetlin, Atropia for epilepsy, - - - - - 573
Taylor, Dr. I. T., Sulphuric acid *vs.* epistaxis, - - 412
Taylor, Peliosis, - - - - - - 276
Tetanus, Treatment, - - - - - - - 276
" Traumatic, - - - - - - 391
Thermo-cautery, a new, - - - - - - 563
Thrombosis, Cerebral, - - - - - - - 49
Thompson, Dr. M. H., Obstetric practice, - - - 534
Thyrotomy, - - - - - - - 1
Tincture of Tayuya, - - - - - - - 94
Transactions of Chicago Med. Soc., - - - 41, 316
Tri-State medical society, Annual meeting, - - - 77
Treatment of wounds, - - - - - - 570
Tuberculosis, meningeal and pulmonary, - - - - 329
" Inoculability, - - - - - - 376
Tumor behind the uterus, - - - - - 289

Turnbull, L., Artificial membrana tympani, - - - 382
Tympanitic resonance, - - - - - 212
Umbilical hernia, Retention of, - - - - 378
Urinary fistula, - - - - - 350
Vaccinal syphilis, - . - - - - 93
Varicose veins, Treatment of, - - - - 567
Version, practiced in Polynesia, - - - - 80
Viability of a six months fœtus, - - - - 469
Viburnum primifolium, - - - - - 92
Vittum, Dr. W. H., Cancer, - - - - 413
Vocal cords, Affections of, - - - - - 175
Voltolini, Prof., Laryngeal polypi, - - - 478
Voss, Communication of syphilis by milk, - - - 93
Wagner, General Pathology, - - - - 551
Wales, Ph. S., Rectal dilator, - - - - - 475
Ward, Whitefield, Hysterical Aphonia, - - - 496
Watson, W. Sp., Diseases of the nose, - - - 133
Weber, Calabar - - - - - 577
Wecker, Use of eserine, - - - - - 578
Weight of new born infants, - - - - 87
Wenzel, Pulsatilla, - - - - - - 576
Wernich, A., Gynæcology in Japan, - - - 415
Whooping-cough, Remedy for, - - - 90, 284
Will, Calculi in the urethra, - - - - 177
Wilson, Dr. E., Healthy skin, - - - - - 262
Windelband, Hemorrhage, - - - - 281
Wiss, Balsam of Peru, - - - - - 183
Wright, Shortening of the limb after fracture of the femur, - 477
Yellow fever, - - - - - - - 359
Ziemssen's Cyclopædia: Diseases of the peripheral cerebro-spinal nerves, - - - - - - - 137
" Diseases of the respiratory organs, - - - 140

### BOOKS AND PAMPHLETS RECEIVED.

Civil Malpractice: A Treatise on Surgical Jurisprudence, etc. By M. A. McClelland, M. D. 1877.

The Cure of Rupture, etc. By George Heaton, M. D., etc. Edited by J. H. Davenport, A. M., M. D., etc. 1877.

Atlas of Skin Diseases. By L. A. Duhring, M. D., etc. Part II. 1877.

Archiv der Pharmacie. VII. Band, 4 Heft.

### ANNOUNCEMENTS FOR THE MONTH.

**MONDAYS.** Societies.
Chicago Medical Society. Regular meetings—*Mondays, June* 4 *and* 18.
Chicago Soc. of Phys. and Surgeons. Regular meetings—*Mondays, June* 11 *and* 25.
Clinics. *Every Monday.*
At Eye and Ear Infirmary, 2 P. M.—Prof. Holmes.
At Central Dispensary (Wood and Harrison sts).—2 P. M. *Gynecological*, Dr. Adolphus; 3 P. M. *Diseases of Children* Dr. R. S. Hall.
At Mercy Hospital—2½ P. M.. *Surgical.* Prof. Andrews.
At Rush College—2¼ P. M., *Medical*, Dr. Bridge.
Lectures. *Every Monday.*
At Rush College (Harrison and Wood sts.)—9 to 1 o'clock, Drs. Wadsworth, Jackson, Danforth and Knox. At Chicago College—8 to 11, Profs. Hatfield, Jewell, and Curtis; 3½, Quine.
At Woman's College—3 to 5, Profs. Paoli and Byford.

**TUESDAYS.** Societies.
Academy of Sciences. Regular meeting at 8 P. M. (263 Wabash av.)—*Tuesday, June* 12.
Clinics. *Every Tuesday.*
At Eye and Ear Infirmary—2 P. M.,Prof. Jones.
At County Hospital—2 P. M., *Medical*. Prof. Johnson. At 3 P. M., *Surgical*, Prof. Gunn.
At Mercy Hospital, 2 P. M. *Medical*, Prof. Hollister.
Lectures. *Every Tuesday.*
At Rush College—9 to 1, Drs. Owens, Bridge, Strong and Case. At Chicago College—9 to 11, Profs. Jewell and Byford.
At Woman's College—9 to 11, Profs. Hayes and Earle.

**WEDNESDAY.** Clinics. *Every Wednesday.*
At County Hospital—2 P. M., *Ophthalmological*, Dr. Montgomery; 3 P. M., *Gynecological*, Prof. Quine.
At Mercy Hospital—2½ P. M., *Ophthalmological*, Prof. Jones.
At Central Dispensary—2 P. M., *Surgery*, Dr. Loomis; *Diseases of Chest*, Dr. Ingals; 3. *Gynecological*. Prof. Etheridge.
Lectures. *Every Wednesday.*
At Rush College—9 to 1, Drs. Wadsworth, Ingals and Sawyer. At Chicago College—9 to 11, Profs. Hyde and Steele; 3½, Jones.
At Woman's College—9 to 11, Prof. Marguerat and Dr. Hale.

**THURSDAYS.** Clinics *Every Thursday.*
At Eye and Ear Infirmary—2 P. M., Prof. Hotz.
At Mercy Hospital—2½ P. M., *Medical*, Prof. Davis.
At Rush College—2 P. M., *Medical*, Prof. Ross; 3 P. M., *Diseases of the Nervous System*, Prof. Lyman.
At Central Dispensary—2 P. M., *Surgical*, Prof. Graham.
Lectures. *Every Thursday.*
At Rush College—9 to 1, Drs. Hayes, Bridge, Strong and Case. At Chicago College—9 to 11, Prof. Quine and Steele; 3½, Byford and Roler.
At Woman's College—9 to 11, Profs. Thompson and Graham.

**FRIDAYS.** Societies.
State Microscopical Society of Illinois. Regular meeting, 8 P. M.—*Friday, June* 8.
Clinics. *Every Friday.*
At County Hospital—2 P. M., *Medical*, Prof. Johnson; 3 P. M., *Surgical*, Prof. Gunn.
At Mercy Hospital—2½ P. M., *Medical*, Prof. Nelson.
At Central Dispensary—2 P. M., *Diseases of Chest*, Dr. Harroun; 3 P. M., *Dermatological* Dr. Maynard.
Lectures. *Every Friday.*
At Rush College—9 to 1, Drs. Wadsworth, Jackson, Strong and Knox. At Chicago. College—8 to 11, Profs. Jones, Hyde, and Curtis; 3½, Hatfield.
At Woman's College—9 to 11, Profs. Bartlett and Blake.

**SATURDAYS.** Clinics. *Every Saturday.*
At Chicago College—1½ P. M.,*Surgical*, Prof. Andrews or Isham; *Gynecological*, Prof. Nelson; 2½ P. M., *Medical*, Prof. Johnson.
At Rush College—2 P. M., *Surgical*, Prof. Gunn.
Lectures. *Every Saturday.*
At Rush College—9 to 1, Drs. Owens, Bridge, Sawyer and Danforth. At Chicago. College—8 to 11, Profs. Merriman, Quine and Byford; 3½, Hyde.
At Woman's College—9 to 11, Dr. Engert.

At all the above named Clinics visiting regular practitioners are, we believe, admitted.

At the South Side Dispensary (Chicago College) there are six daily special Clinics, for sections of the classes of the Chicago College.

THE CHICAGO

### Medical Journal and Examiner.

EDITOR: WILLIAM H. BYFORD, A.M., M.D.

ASSOCIATE EDITORS: JAMES H. ETHERIDGE, M.D.; NORMAN BRIDGE, M.D.; JAS. NEVINS HYDE, A.M., M.D.; FERD. C. HOTZ, M.D.

ASSISTANT EDITOR: E. FLETCHER INGALS, M. D.

TERMS—FOUR DOLLARS PER ANNUM, IN ADVANCE, POSTAGE FREE.

*Subscribers and Friends of the Chicago Medical Journal and Examiner:*

About seventy of the leading physicians of this city, in the month of August, 1875, organized the MEDICAL PRESS ASSOCIATION, for the purpose of founding a medical library. As an auxiliary to the library, the association undertook the editorial management of the CHICAGO MEDICAL JOURNAL AND EXAMINER, a periodical which was to take the place of the Chicago Medical Joarnal, which had been purchased, and the Medical Examiner, which was generously donated to the association.

At that time Messrs. Keene, Cook & Co. held a contract for the publication of the Chicago Medical Journal, which they did not wish to relinquish; therefore the new journal was placed in their hands, with the arrangement that they should bear all the expenses and retain all the profits of its publication. They agreed to turn over to the association all exchanges and new books for review. This arrangement continued until Jan. 20, 1877, when the stringency of the times forced Keene, Cook & Co. into bankruptcy.

This misfortune threw the responsibility of the publication of the journal on the association. A large amount of money which had been collected on the current year prior to Jan. 20, went with the assets of the firm to satisfy their creditors, and was thus lost to the association. However, the journal will be continued, without interruption, to *all* subscribers. The journal is issued monthly, and contains for the year 1,152 closely printed pages.

The editors have secured some of the best talent in the country to contribute origina articles, and every month they receive more than a hundred of the leading medical journals of Great Britain, Ireland, France, Spain, Portugal, Italy, Prussia, Russia, Austria, Denmark, Australia, South America, Mexico, and the United States and Canada, from which they cull items of special interest. They give careful attention to the review of new books, and thus they furnish their readers all the current medical literature of the day.

We feel confident that the journal cannot fail to recommend itself to every careful reader, and we assure our friends that its rank will be maintained with the best periodicals in the country. In future, all the profits which may accrue from its publication will be devoted to the improvement of the library.

E. FLETCHER INGALS, ASSISTANT EDITOR,
188 Clark street, Chicago.

